# Improvement of Rheumatic Valvular Heart Disease in Patients Undergoing Prolonged Antibiotic Prophylaxis

**DOI:** 10.3389/fcvm.2021.676098

**Published:** 2021-06-23

**Authors:** Renato Pedro de Almeida Torres, Rômulo Francisco de Almeida Torres, Gabrielle de Crombrugghe, Scarllet Palacin Moraes da Silva, Sarah Leticia Veroneze Cordeiro, Karine Alessandra Bosi, Pierre R. Smeesters, Rosângela Stadnick Lauth de Almeida Torres

**Affiliations:** ^1^Department of Pediatric Cardiology, Hospital Pequeno Principe, Curitiba, Brazil; ^2^Department of Hemodynamics and Interventional Cardiology, Hospital Marcelino Champagnat, Curitiba, Brazil; ^3^Division of Pediatric Infectious Diseases and Infection Prevention and Control, Hospital Universitaire des Enfants Reine Fabiola, Brussels, Belgium; ^4^Department of Medicine, Universidade Positivo, Curitiba, Brazil; ^5^Universidade Positivo, Curitiba, Brazil; ^6^Murdoch Childrens Research Institute, Royal Children's Hospital, Melbourne, VIC, Australia; ^7^Department of Paediatrics, University of Melbourne, Melbourne, VIC, Australia; ^8^Molecular Bacteriology Laboratory, Faculty of Medicine, Free University of Brussels, Brussels, Belgium; ^9^Epidemiology Laboratory and Disease Control Division, Laboratório Central do Estado do Paraná, Curitiba, Brazil

**Keywords:** secondary prophylaxis, rheumatic heart disease, benzathine penicillin G, Group A β-hemolytic *Streptococcus*, carditis, recurrence

## Abstract

Secondary prophylaxis of rheumatic heart diseases is efficient in reducing disease recurrence, heart damage, and cardiac impairment. We aimed to monitor the clinical evolution of a large Brazilian cohort of rheumatic patients under prolonged secondary prophylaxis. From 1986 to 2018, a cohort of 593 patients with rheumatic fever was followed every 6 months by the Reference Center for the Control and Prevention of Rheumatic Fever and Rheumatic Cardiopathy (CPCFR), Paraná, Brazil. In this cohort, 243 (41%) patients did not present cardiac damage (group I), while 350 (59%) were diagnosed with rheumatic heart disease (RHD) (group II) using the latest case definition. Among group II, 233 and 15 patients had impairment of the mitral and aortic valves, respectively, while 102 patients had impairment of both valves. Lesions on the mitral and aortic valves presented a regression in 69.9 and 48.7% of the patients, respectively. Active patient recruitment in the reference center and early detection of oropharyngeal GAS were important factors for optimal adherence to the prophylactic treatment. Patients with disease progression were associated with noncompliance to secondary prophylaxis. No patients undergoing regular prophylaxis presented progression of the rheumatic cardiac disease. Eighteen valvular surgeries were performed, and four (0.7%) patients died. This study confirmed that tailored and active efforts invested in rheumatic heart disease secondary prevention allowed for significant clinical improvement.

## Introduction

Acute rheumatic fever (ARF) is an inflammatory, autoimmune disease induced by a throat infection caused by *Streptococcus pyogenes* (Group A β-hemolytic *Streptococcus*—GAS) in genetically predisposed individuals. Global prevalence of rheumatic heart disease (RHD) is estimated to be 33.4 million and is responsible for about 319,400 deaths per year (1). Most cases of ARF occur in low- and middle-income countries where limited resources are often available for optimal health programs. In Brazil, a significant amount of financial resources from the Brazilian Unified Health System (SUS) is intended to assist and treat ARF and RHD patients (2, 3).

Promising progresses have been recently made toward a safe and protective vaccine against streptococcal infections (4, 5). However, prophylaxis remains, so far, the most effective treatment option to prevent RHD recurrences. Benzathine penicillin-based prophylaxis, every 3–4 weeks, remains the treatment of choice since GAS continues to be fully susceptible to penicillin (6, 7).

Secondary prophylaxis of RHD is known for modifying the natural history of the disease, allowing for the prevention of disease recurrence and consequently the prevention of further development of heart damage and/or cardiac impairment (8–10). Nevertheless, less is known about the impact of secondary prophylaxis on the recovering of cardiac damage in patients who regularly undergo a prolonged prophylactic treatment. The aim of this study is to describe the RHD secondary prevention program in the Brazilian state of Paraná and monitor the cardiac evolution of rheumatic patients undergoing prolonged and regular secondary prophylaxis.

## Materials and Methods

This prospective cohort study monitors the clinical evolution of patients with confirmed ARF diagnosed on the basis of the 2015 revised Jones criteria (11). The patients were diagnosed, registered, and followed up at the Center of Reference for the Control and Prevention of Rheumatic Fever and Rheumatic Cardiopathy (CPCFR) of the Health Secretariat of the State of Paraná/Brazil from July 1986 through June 2018.

All included patients had a confirmed history of ARF or presented with one or more morphological features of RHD according to the World Heart Federation (WHF) criteria (12). Doppler and morphological echocardiogram findings are detailed in Supplementary Table 2.

RHD patients were classified according to the severity of their valvulopathy into mild, moderate, or severe according to the most recent definition (13, 14). Patients with borderline RHD according WHF guidelines (12), those who remained under prophylaxis treatment for <2 years, and those receiving oral penicillin treatment were excluded from the study.

Patients were divided into two groups. Group I included patients without cardiac damage, and group II included patients who presented cardiac damage consistent with RHD, either as an isolated manifestation or in association with arthritis, chorea, erythema marginatum, and subcutaneous nodules (Supplementary Table 1).

All patients were invited to follow a secondary antibiotic prophylaxis using intramuscular (IM) benzathine penicillin G every 21 or 28 days as recommended by the American Heart Association (AHA) (6) and to undergo a follow-up clinical and echocardiographic evaluation every 6 months. To improve compliance, reminder messages were sent to those who missed their semester consultation. Throat swab was performed at each consultation searching for the presence of GAS. In case of positive culture, discussion was undertaken with the patients to recall the importance of treatment compliance.

Adherence to the prophylactic treatment was checked at each consultation during follow-up. The patients receiving penicillin injections every 21 days were considered as compliant and included in the “regular prophylactic treatment group” when receiving at least 13 doses/year (more than 76% of the doses), with a maximum delay of 7 days, i.e., 28 days between the doses. Patients who received <13 doses/years and/or had spaced their doses (more than 28 days between doses) were included in the “irregular prophylactic treatment group.”

The patients receiving penicillin injections every 28 days were included in the “regular prophylactic treatment group” when receiving at least 10 doses/years (more than 76% of the doses), with a maximum delay of 7 days, i.e., 35 days between the doses. Patients who received <10 doses/years and/or had spaced their doses (more than 35 days between doses) were included in the “irregular prophylactic treatment group.”

The progression of the cardiac impairment was assessed by comparing the type and severity of valvular heart damage when the patient first registered at the CPCFR with the type and severity of the valvular heart damage at discharge. Progression was defined as a change in diagnostic (RHD or normal) or a modification in the severity of RHD cases (mild, moderate, or severe cases).

To determine possible associations between the categorical variables, chi-square (χ^2^) and *t* tests were used to compare means; correction was performed by Fisher's exact test. The significance level of *p* < 0.05 was adopted.

This study was approved by the Research Ethics Committee at the Pequeno Príncipe Hospital under CAAE-02153912.2.0000.0097.

## Results

This study has monitored 593 ARF patients from the 709 registered at the CPCFR during the study period. One hundred and sixteen patients (16.4%) were excluded for not meeting the inclusion criteria: 70 (9.9%) for not complying with at least 2 years of follow-up, 26 (3.7%) for presenting borderline RHD, and 20 (2.8%) for oral penicillin V prophylaxis. Three hundred and nine patients (52.1%) were female and 284 (47.9%) were male. Patients included in the study were between 2 and 21 years old at the beginning of follow-up. The follow-up time ranged from 2 to 26 years with almost half of the patients (47.6%) followed for more than 11 years (Table 1).

**Table 1:** Rheumatic fever patients' characteristics (group I and II).

**Characteristic *N* (%)**	
**Gender**	
Female	309 (52.1)
Male	284 (47.9)
Age at screening, years	2–21 years (median of 9 years)
Duration of follow-up, years	2–26 years (median of 10 years)
**Duration of follow-up detailed:**	
2 to <3 years: 11 (1.9%)	
3 to <5 years: 70 (11.8%)	
5 to <6 years: 32 (5.4%)	
6 to <10 years: 165 (27.8%)	
10 to <11 years: 31 (5.2%)	
≥11 years: 284 (47.9%)	
**Time of follow-up necessary to achieve total recovery of valve lesions:**	
Recovery of mitral valve (*N =* 104):	Recovery of aortic valve (*N =* 10):
After 2 years = 40 (38.5)	After 2 years = 5 (50.0)
After 5 years = 53 (50.9)	After 5 years = 5 (50.0)
After >5 years = 11 (10.6)	After >5 years = 0 (0.0)

All patients started their prophylactic treatment with IM penicillin every 3 weeks, as recommended by the AHA for populations with particularly high incidence of rheumatic fever (6). A minority of the patients, 62 (10.4%), spaced their prophylactic treatment to every 4 weeks when they completed 21 years of age. GAS isolation in the culture of the patients' oropharynx helped identify non-compliance to the secondary prophylaxis in 92 (15.5%) patients. These patients received more attention and guidance from the CPCFR team to raise their awareness about the risks of recurrence and progression of the disease.

**Figure 1:**
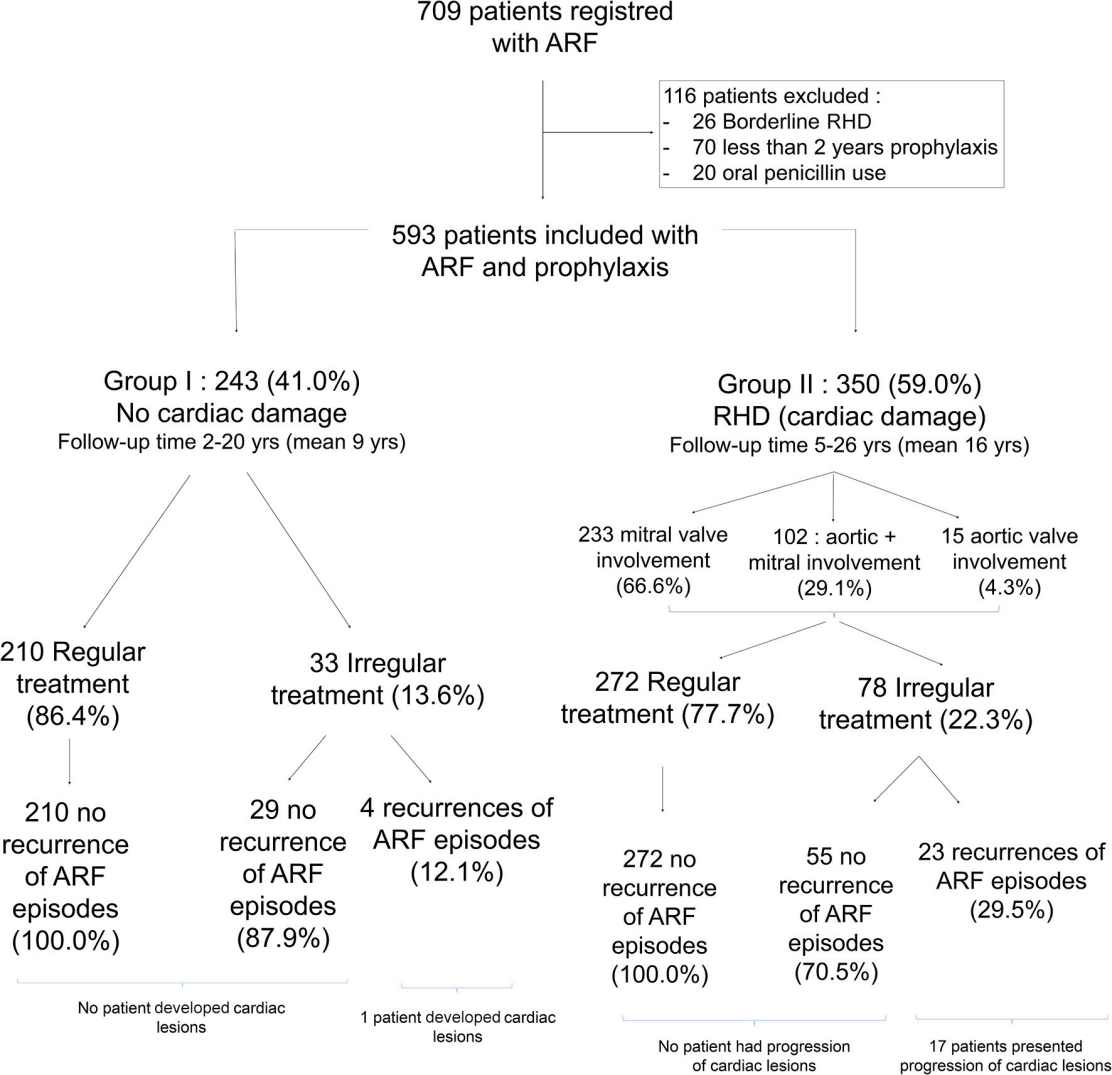
Follow-up of 613 rheumatic patients undergoing prolonged secondary prophylaxis.

Group I consisted of 243 patients (41.0%) without cardiac lesions. At the beginning of the follow-up, 207 of these patients (85.2%) had presented a single episode of ARF, and 36 (14.8%) had presented more than one episode. The follow-up time of this group ranged from 2 to 20 years (median of 9 years). Most patients (210; 86.4%) were compliant to prophylaxis and included in the “regular prophylactic treatment group,” while 33 (13.6%) were not always compliant and therefore included in the “irregular prophylactic treatment group.” Four patients (12.1%) from group I had a recurrence of ARF: two with arthritis, one presented two recurrences of chorea, and one developed carditis and mild mitral regurgitation (MR). All these episodes of recurrences occurred in the “irregular prophylactic treatment group” (Figure 1).

Group II consisted of 350 patients (59.0%) with cardiac lesions consistent with RHD (12). At the start of the follow-up, 206 patients (58.9%) had presented a single episode of ARF, while 144 (41.1%) had presented more than one episode. Most patients in group II presented mitral valve involvement (233; 66.6%), 15 patients (4.3%) presented aortic valve involvement, and 102 patients (29.1%) presented coexistence of mitral and aortic involvement (Table 2). The follow-up time of this group ranged from 5 to 26 years of age (median of 16 years). Two hundred and seventy-two patients (77.7%) were included in the “regular prophylactic treatment group” while 78 (22.3%) underwent irregular prophylactic treatment. In the irregular treatment group, we observed 23 (29.5%) recurrences of ARF episodes, leading to a worsening of the cardiac lesions in 17 patients (Figure 1). Gender analyses of mitral and aortic lesion evolution did not show significant differences (Table 3). Although recurrences were observed in all age groups, the highest frequency, even if age difference is not statistically significant, was overall observed among adolescents (14 to 16 years old) (Table 3).

**Table 2:** Group II: evolution of the cardiac impairment in 350 patients with rheumatic heart disease undergoing prophylactic treatment.

		**Diagnosis at follow-up N (%)**										
**Diagnosis at baseline N (%)**		**Total improvement**		**Partial improvement**		**Unchanged**		**Progression**		**Surgeries**		**Death**
		**Mitral**	**Aortic**	**Mitral**	**Aortic**	**Mitral**	**Aortic**	**Mitral**	**Aortic**	**Mitral**	**Aortic**
**Mitral valve involvement**												
MR^a^	218 (62.3)	93 (42.7)		59 (27.1)		56 (25.7)		8 (3.7)		2 (0.9)		3 (1.4)
MS^b^	3 (0.9)					1 (33.3)				2 (66.7)	
DM^c^	12 (3.4)			4 (33.3)		1 (8.3)		1 (8.3)		6 (50.0)	
Sub-Total	233 (66.6)											
**Aortic valve involvement**												
AR^d^	15 (4.3)		2 (13.3)		1 (6.7)		9 (60.0)		1 (6.7)		2 (13.3)
Sub-Total	15 (4.3)											
**Mitral valve involvement**												
MR and AR	98 (28.0)	11 (11.2)	8 (8.2)	66 (67.3)	43 (43.8)	13 (13.3)	45 (45.9)	4 (4.1)	2 (2.0)	4 (4.1)		1 (1.0)
MS and AR	2 (0.6)				1 (50.0)	1 (50.0)				1 (50.0)	1 (50.0)
MR and DAL^e^	2 (0.6)			1 (50.0)	2 (100.0)			1 (50.0)			
Sub-Total	102 (29.1)											
**Total Mitral valve involvement**		104 (31.0)		130 (38.8)		72 (21.5)		14 (4.2)		15 (45)		3 (1.4)
**335 (95,7)**											
**Total Aortic valve involvement**			10 (8.5)		47 (40.2)		54 (46.2)		3 (2.6)		3 (2.6)	1 (1.0)
**117 (33,4)**											

a *MR, Mitral Regurgitation*;

b *MS, Mitral Stenosis*;

c *DML, Double Mitral Lesion (MS + MR)*;

d *AR, Aortic Regurgitation*;

e *DAL, Double Aortic Lesion (AS + AR)*.

**Table 3:** Evolution of mitral and aortic lesions in group 2 (*N* = 350).

	**Mitral lesions (*****N=*** **335)**				**Aortic lesions (*****N=*** **117)**			
	**Number of patients**	**Number (%) of cardiac lesion regression (partial or complete)**	**Number (%) of cardiac lesion unchanged or worsened**	***p*-value**	**Number of patients**	**Number (%) of cardiac lesion regression (partial or complete)**	**Number (%) of cardiac lesion unchanged or worsened**	***p-*value**
Female	174	121 (69.5)	53 (30.5)	0.905	60	28 (46.7)	32 (53.3)	0.712
Male	161	113 (70.2)	48 (29.8)		57	29 (50.9)	28 (49.1)	
Age at disease onset				0.161				0.579
0–4 years old	29	24 (82.8)	5 (17.2)		0	0	0	
5–9 years old	189	132 (69.8)	57 (30.2)		62	32 (51.6)	30 (48.4)	
10–14 years old	116	78 (67.2)	38 (32.8)		54	25 (46.2)	29 (53.7)	
≥15 years old	1	0	1 (100.0)		1	0	1 (100)	

*Of note, the same patient may present with different valves lesions. Fisher's exact test was used to analyse differences between sex and age at diagnostic*.

Many patients from group II benefitted from the secondary prophylaxis. The patients presenting aortic regurgitation and/or mitral regurgitation were those who benefitted most from regular prophylaxis, presenting the highest proportion of recovery of their valve lesions. Two hundred thirty-four patients (69.9%) presented regression of the mitral valve lesions including 104 (31.0%) with a total regression. Fifty-seven patients (48.7%) presented regression of the aortic valve lesions including 10 (8.5%) with a total regression and 47 (40.2%) with a partial regression. The regeneration of the mitral valve was significantly higher than the aortic valve regeneration (*p* < 0.001) (Tables 1, 2). Forty (38.5%) patients of the 104 with mitral valve recovery were observed after 5 years of follow-up, whereas half of the 10 with aortic valve recovery were observed after only 2 years of follow-up (Table 1). Although some patients presenting mitral stenosis (MS), double mitral lesion (DML), mitral stenosis combined with aortic regurgitation, as well as double aortic lesion (DAL) combined with mitral regurgitation have benefitted from the secondary prophylaxis, none of these patients achieved total regression of the preexisting lesions. Nine (50%) of the 18 surgeries performed were done on these patients. The 17 patients presenting recurrence of ARF episodes resulting in progression of their valve damage were part of the irregular prophylactic treatment group. No patients undergoing the regular prophylactic treatment presented recurrence of ARF, and none of them presented progression of the valve damage. Four patients (1.1%) with irregular prophylaxis died (Table 2).

## Discussion

To our knowledge, this study describes the largest cohort of rheumatic patients undergoing secondary prophylaxis and the longest follow-up time. The 593 patients were followed up for up to 26 years, with almost half of the patients being followed for more than 11 years, with clinical, microbiological, and echocardiographic monitoring.

Although ARF and RHD represent a significant matter of concern for low- and middle-income countries, few countries have efficient prevention program in place. The REMEDY study (Global Rheumatic Heart Disease Registry) reports this reality. The lack of efficient prevention program leads to patients being unfortunately diagnosed at an advanced stage of cardiac valve disease too often presenting pulmonary hypertension and/or other complications (15). A similar situation has been described in Fiji, where only 6.3% of the patients with RHD received ≥80% of the prescribed injections and only 2% of the patients received regular antibiotic prophylaxis (16). Without prophylaxis, the disease evolves rapidly leading to hospitalization and surgical intervention.

When a prevention program is in place, one of the key goals is to obtain, and maintain, the patient's adherence to secondary prophylaxis (1, 17, 18). Usual obstacles to strong secondary prophylaxis program include difficulties related with the patient's registration, recording of injection dates, injection-associated pain, lack of IM penicillin in remote health centers, and limited training of the health care workers (19–21). According to Dassel et al., some protection is already provided to patients who receive 40% of the prescribed doses for secondary prophylaxis; however, a lower percentage (<20%) of doses is associated with a fourfold increase in the odds of having a recurrence (22). The proportion of regular treatment adherence was relatively high in our study (81.3%). Program prioritization and significant human resource dedicated to such program is likely to play an instrumental role for this overall good adherence. Although we could not find an epidemiological report confirming our assertion, our personal experience suggests that the interval's extension between doses (4 weeks rather than 3 weeks) may have convinced some patients not to abandon their prophylaxis treatment. Additionally, detection of oropharyngeal GAS was an important tool for maintaining optimal adherence on the long term.

None of the patients undergoing regular prophylactic treatment presented recurrence of ARF episodes or progression of the heart disease. On the contrary, significant rates of ARF recurrence were observed among patients who received irregular prophylactic treatment (12.1 and 29.5% in groups I and II, respectively). In comparison, the cumulative incidence of recurrences identified in Australian indigenous communities with a low rate of adherence to secondary prophylaxis was 3.8% in the 1st year, 14.9% in the 5th year, and 20.1% in the 10th year of the study (23). Approximately 50% of the Australian indigenous RHD population required surgery within 2 years and 10% died after 6 years of their initial diagnosis (18).

Adherence to secondary prophylaxis also resulted in cardiac recovery in a significant number of patients, including some discharged with no echocardiographic abnormalities. Cases with follow-up interval longer than 5 years were more likely to improve. In our study, the patients who most benefitted from the secondary prophylaxis were those who did not present carditis at the start of the treatment (Group I). A study by Haran et al. reports that four of the six patients with no initial valvular involvement developed valvular alterations during the 27-months follow-up study; half of them were compliant to secondary prophylaxis (24). Even if resistance to penicillin is not a concern for GAS so far, the potential long-term effect of prolonged antibiotic prophylaxis should be monitored.

In our study, patients who presented rheumatic cardiac lesions (Group II) also benefitted from secondary prophylaxis. Total or partial regression of the cardiac lesions was observed in 69.8 and in 48.7% of the patients with mitral and aortic damage, respectively. Regeneration of the mitral valve (the most frequent lesion in our patients) was significantly higher than the aortic valve. Previous studies also shown that cardiac impairment improved in 43.5–51% of the patients undergoing prophylactic treatment, with the highest improvement for mitral regurgitation (>70% improvement) (8–10). In Northern Australia, 17 RHD patients were monitored, with a 70% rate of adherence to secondary prophylaxis; five patients presented total regression, two partial regression, seven kept their former lesions, and three were reported with progression of their valvular disease (24). In Pakistan, 21 RHD patients were monitored for 10 years; among them, only six adhered to secondary prophylaxis, presenting regression of their preexisting valvular lesions. The other patients were reported with progression in the severity of their first lesions or with development of new valvular lesions with a high death rate (23%) (25). In Brazil, 462 rheumatic patients were followed up for 13.6 years. More than one third of them presented recurrences by non-adherence to secondary prophylaxis (26).

Our results from Brazil shows that dedicated efforts for secondary prevention of ARF and RHD allow for significant clinical improvement. Close follow-up including clinical, microbiological, and echocardiographic monitoring is needed for a prolonged period of time to reach that goal.

## Data Availability Statement

The original contributions presented in the study are included in the article/Supplementary Material, further inquiries can be directed to the corresponding author/s.

## Ethics Statement

The studies involving human participants were reviewed and approved by Pequeno Príncipe Hospital, under CAAE-02153912.2.0000.0097. Written informed consent to participate in this study was provided by the participants' legal guardian/next of kin.

## Author Contributions

RPAT and RSLAT conceived the project. RPAT, RSLAT, and RFAT conducted the study according to the protocol. RPAT, RSLAT, RFAT, PRS, and GC analyzed the results. RPAT, RSLAT, RFAT, PRS, GC, SPMS, SLVC, and KAB wrote and reviewed the manuscript. All authors contributed to the article and approved the submitted version.

## Conflict of Interest

The authors declare that the research was conducted in the absence of any commercial or financial relationships that could be construed as a potential conflict of interest.

## Supplementary Material

Supplementary Table 1 Most prevalent manifestations presented by 593 patients at the start of the follow-up. ^*^ARF, Acute rheumatic fever; ^**^EM, Erythema Marginatum; ^***^SN, Subcutaneous Nodules.

Supplementary Table 2 Criteria for the diagnosis of RHD patients^*^. ^*^Gewitz et al. (11).
