# Supplementary material for: Improvement of Rheumatic Valvular Heart Disease in Patients Undergoing Prolonged Antibiotic Prophylaxis
Source: Front Cardiovasc Med. 2021 Jun 23;8:676098. doi: 10.3389/fcvm.2021.676098 (PMC8260676; doi:10.3389/fcvm.2021.676098)
Supplement: Supplementary Table 1 — Most prevalent manifestations presented by 593 patients at the start of the follow-up. *ARF, Acute rheumatic fever; **EM, Erythema Marginatum; ***SN, Subcutaneous Nodules. [file Table_1.pdf]

Supplementary data: Table S1. Most prevalent manifestations presented by 593 patients at the start of the follow-up.

	Benzathine penicillin every 21 days (n. 531)				Benzathine penicillin every 28 days (n. 62)				
	Group I		Group II		Group I		Group II		Total
More Prevalent Manifestations	Single episode of ARF*	> 1 episode of ARF	Single episode of ARF	> 1 episode of ARF	Single episode of ARF	> 1 episode of ARF	Single episode of ARF	> 1 episode of ARF	n (%)
Arthritis	181	28	-	-	-	-	-	-	209 (35.2)
Chorea	24	6	-	-	-	-	-	-	30 (5.1)
Arthritis/Chorea	2	2	-	-	-	-	-	-	4 (0.7)
Carditis	-	-	46	43	-	-	14	12	115 (19.4)
Carditis/Arthritis	-	-	89	49	-	-	13	13	164 (27.7)
Carditis/Chorea	-	-	21		-	-	2	-	31 (5.2)
Carditis/Arthritis/Chorea	-	-	13	12	-	-	3	5	33 (5.7)
Carditis/EM	-	-	1	-	-	-	-	-	1 (0.2)
Carditis/Chorea/EM**	-	-	1	-	-	-	-	-	1 (0.2)
Carditis/Arthritis/EM	-	-	-	1	-	-	-	-	1 (0.2)
Carditis/Arthritis/EM/SN***	-	-	1	-	-	-	-	-	1 (0.2)
Carditis/Arthritis/Chorea/EM	-	-	1	-	-	-	-	-	1 (0.2)
Carditis/Arthritis/SN	-	-	1	1	-	-	-	-	2 (0.3)
Total	207	36	174	114	0	0	32	30	593

*ARF - Acute rheumatic fever, **EM - Erythema Marginatum, ***SN - Subcutaneous Nodules
