# Supplementary material for: Improvement of Rheumatic Valvular Heart Disease in Patients Undergoing Prolonged Antibiotic Prophylaxis
Source: Front Cardiovasc Med. 2021 Jun 23;8:676098. doi: 10.3389/fcvm.2021.676098 (PMC8260676; doi:10.3389/fcvm.2021.676098)
Supplement: Supplementary Table 2 — Criteria for the diagnosis of RHD patients*. *Gewitz et al. (11). [file Table_2.docx]

Supplementary table S2: Criteria for the diagnosis of RHD patients*.

| Doppler Findings (all 4 criteria met) | |
| --- | --- |
| **Pathological mitral regurgitation** | **Pathological aortic regurgitation** |
| - Seen in at least 2 views | - Seen in at least 2 views |
| - Jet length ≥2 cm in at least 1 view | - Jet length ≥1 cm in at least 1 view |
| - Peak velocity >3 m/s | - Peak velocity >3 m/s |
| - Pansystolic jet in at least 1 envelope | - Pan diastolic jet in at least 1 envelope |
|  |  |
| Morphological Findings on Echocardiogram | |
| **Mitral valve changes** | **Aortic valve changes** |
| - Annular dilation | - Irregular or focal leaflet thickening |
| - Chordal elongation | - Coaptation defect |
| - Chordal rupture resulting in flail leaflet with severe mitral regurgitation | - Restricted leaflet motion - Leaflet prolapse |
| - Anterior (or less commonly posterior) leaflet tip prolapse |  |
| - Beading/nodularity of leaflet tips |  |
| - Chronic mitral valve changes: not seen in acute carditis |  |
| - Leaflet thickening |  |
| - Chordal thickening and fusion |  |
| - Restricted leaflet motion |  |
| - Calcification |  |

*Gewitz, 2015.
